# Micro/Nanorobotic Systems for Imaging-Guided Closed-Loop Thrombus Recanalization

**DOI:** 10.34133/cbsystems.0592

**Published:** 2026-06-05

**Authors:** Jiajun He, Zhixin Xia, Lipeng Liao, Xu Li, Xuhao Wu, Jie Shen, Qinglong Wang, Ben Wang

**Affiliations:** ^1^ College of Chemistry and Environmental Engineering, Shenzhen University, Shenzhen 518055, China.; ^2^ Department of Spine Surgery, Peking University Shenzhen Hospital, PKU-Shenzhen Clinical Institute of Shantou University Medical College, Shenzhen 518036, China.; ^3^ Shenzhen Key Laboratory of Spine Surgery, Department of Spine Surgery, Peking University Shenzhen Hospital, Shenzhen 518036, China.; ^4^ Department of Mechanical and Automation Engineering, The Chinese University of Hong Kong, Hong Kong SAR, China.

## Abstract

How can we reopen blocked blood vessels quickly and safely when a clot forms in stroke, heart attack, deep-vein thrombosis, or pulmonary embolism? Current care relies on clot-dissolving drugs given through the bloodstream and catheter-based procedures that mechanically retrieve thrombi and/or deliver drugs locally. These methods can be lifesaving, but they are limited by residual distal microvascular obstruction, reduced efficacy against dense or lysis-resistant clots, and procedure-related complications such as hemorrhage and downstream embolization. This review compares 2 complementary technology paths: “tethered” catheter systems designed for rapid and operator-controlled reopening of large vessels, and “untethered” micro/nanoscale systems, including injectable carrier-based platforms and actively actuated micro/nanorobots, that can be actuated and guided by magnetic, ultrasound, or light energy to seek, penetrate, and disrupt clots at the microscale. Across reported studies, rationally engineered nanocarriers can improve clot targeting and local retention of thrombolytic payloads, while externally powered microrobots and microswarms enable active mixing and mechanical assistance, often reducing treatment time and lowering required drug doses in preclinical models. We further summarize imaging and tracking strategies that ensure feedback control of device position, energy delivery, and treatment endpoints. Finally, we discuss key translational barriers, including robust navigation under complex hemodynamics, reliable stopping and clearance, standardized safety windows, and seamless integration into established interventional workflows, thereby outlining actionable priorities that can steer this field toward clinical therapies.

## Introduction

**Fig. 1.**
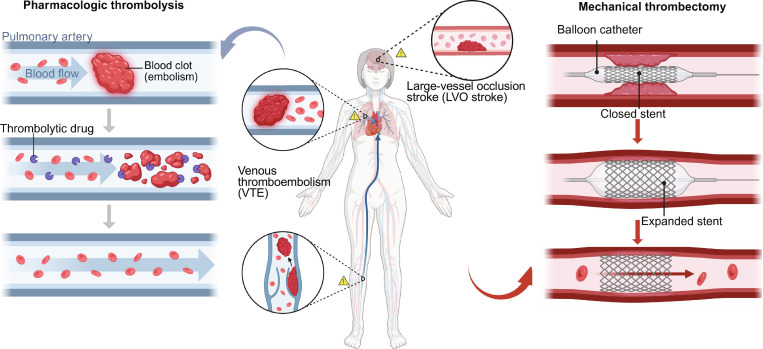
Common thrombotic occlusions in the human body and 2 major recanalization modalities: tethered endovascular intervention (right) and untethered micro/nanoagent-based therapy (left). Figure created with BioRender.com.

Thrombosis represents a fundamental pathological process underlying a wide spectrum of cardiovascular, cerebrovascular, and venous thromboembolic diseases, with profound and often devastating clinical consequences [1–3]. Whether arising as arterial thrombi that precipitate myocardial infarction and ischemic stroke or as venous thrombi responsible for pulmonary embolism and deep vein thrombosis, the core pathophysiological mechanism involves the abnormal intravascular coagulation of blood and subsequent formation of an occlusive thrombus, thereby obstructing downstream perfusion. Such obstruction leads directly to tissue ischemia and hypoxia and, in severe cases, may rapidly progress to life-threatening organ dysfunction [4,5]. Consequently, timely restoration of vessel patency together with effective distal reperfusion remains a decisive determinant of mortality and disability reduction in clinical management. However, in routine clinical practice, achieving precise localization of the thrombus, efficient clearance, and durable vascular recanalization demands exceptionally high standards of speed, precision, and procedural robustness [6–11]. These demands strain not only clinical decision-making and operator skill but also the performance limits of current recanalization technologies and devices. At present, the prevailing clinical strategies for vascular recanalization can be broadly categorized into 2 major approaches: pharmacological thrombolysis and tethered endovascular interventions [12–17] (Fig. 1). Although these approaches constitute the cornerstone of current clinical thrombus management, their inherent limitations have become increasingly evident when confronted with the complexity and heterogeneity of clinical scenarios.

Thrombosis represents a fundamental pathological process underlying a wide spectrum of cardiovascular, cerebrovascular, and venous thromboembolic diseases, with profound and often devastating clinical consequences [1–3]. Whether arising as arterial thrombi that precipitate myocardial infarction and ischemic stroke or as venous thrombi responsible for pulmonary embolism and deep vein thrombosis, the core pathophysiological mechanism involves the abnormal intravascular coagulation of blood and subsequent formation of an occlusive thrombus, thereby obstructing downstream perfusion. Such obstruction leads directly to tissue ischemia and hypoxia and, in severe cases, may rapidly progress to life-threatening organ dysfunction [4,5]. Consequently, timely restoration of vessel patency together with effective distal reperfusion remains a decisive determinant of mortality and disability reduction in clinical management. However, in routine clinical practice, achieving precise localization of the thrombus, efficient clearance, and durable vascular recanalization demands exceptionally high standards of speed, precision, and procedural robustness [6–11]. These demands strain not only clinical decision-making and operator skill but also the performance limits of current recanalization technologies and devices. At present, the prevailing clinical strategies for vascular recanalization can be broadly categorized into 2 major approaches: pharmacological thrombolysis and tethered endovascular interventions [12–17] (Fig. 1). Although these approaches constitute the cornerstone of current clinical thrombus management, their inherent limitations have become increasingly evident when confronted with the complexity and heterogeneity of clinical scenarios.

Pharmacological thrombolysis, particularly systemic intravenous administration, remains one of the most widely adopted recanalization strategies in current clinical practice. Its mechanism relies on intravenously delivered fibrinolytic agents (e.g., alteplase and urokinase) to activate the endogenous fibrinolytic cascade and dissolve the obstructive thrombus. However, this “systemic delivery with localized action” paradigm is constrained by 2 intrinsic limitations [6–11]. First, thrombolytic agents exhibit exceedingly poor target specificity. Once entering the circulation, these agents depend solely on passive convection to reach the occlusion site, during which their concentration becomes substantially diluted throughout the bloodstream, resulting in markedly insufficient drug levels and suboptimal lytic efficacy at the thrombus region. Second, systemic drug exposure introduces important safety concerns, as fibrinolytics may simultaneously disrupt physiological hemostasis, thereby increasing the risk of severe and sometimes fatal hemorrhagic complications (e.g., intracranial and gastrointestinal bleeding). This risk profile narrows eligibility in older patients, those with bleeding tendencies or recent surgical history, necessitating a continual trade-off between anticipated reperfusion benefit and potentially life-threatening hemorrhage.

Tethered endovascular interventions, including catheter-directed thrombolysis (CDT) and percutaneous mechanical thrombectomy (PMT), deliver catheters, guidewires, and adjunct devices intraluminally to the thrombus, enabling highly localized “point-to-point” treatment that effectively overcomes the poor target specificity of systemic thrombolysis. These techniques can rapidly restore patency in large vessels and have become indispensable for acute large-vessel occlusion management [18–24]. Despite these advantages, tethered interventions face several fundamental and often difficult-to-overcome limitations. The most prominent are anatomical constraints, as the diameter, flexibility, and navigability of catheters and guidewires limit their ability to access tortuous or narrow territories, such as distal intracranial branches or microcirculation. Moreover, their efficacy is also compromised when treating dense or chronic thrombi. Mechanical fragmentation or aspiration may dislodge thrombus debris, which can migrate distally and block microvessels, resulting in new “no-reflow” zones within the microcirculation. In addition, many catheter-based techniques (e.g., CDT) still depend on the intact fibrinolytic system to degrade the thrombus. As a result, thrombi that are highly cross-linked, fibrin-rich, or biochemically resistant remain difficult to dissolve even with local drug delivery. Taken together, these limitations prevent tethered interventions from achieving complete and durable reperfusion, especially at the microvascular regions where residual obstruction and perfusion deficits frequently occur.

Recent advances in micro/nanorobotic systems have highlighted a broader trend toward structurally integrated and functionally specialized therapeutic platforms, including modular nanorobots that combine targeting, propulsion, and microenvironment-responsive capabilities within a single architecture [25]. Confronted with the inherent limitations of conventional recanalization strategies, the rapid advances in nanomaterials science, micro/nanofabrication technologies, and bioinspired engineering have introduced transformative concepts and tools to the field of thrombus management. The convergence of these frontier technologies has given rise to 2 parallel yet fundamentally distinct innovation pathways. The first builds upon existing catheter-based platforms by integrating micro/nanoscale therapeutic agents or energy input units to develop hybrid “tethered + micro/nano” recanalization systems. The second departs entirely from catheter-dependent methods, instead leveraging injectable micro/nanoscale agents capable of truly untethered, remotely actuated, and highly targeted thrombolysis [18–24]. Representative studies have further shown that externally powered or nanozyme-integrated nanomotors can actively enhance local accumulation, tissue penetration, and synergistic therapeutic output, illustrating the potential of untethered systems beyond passive carrier-based delivery [26]. These pathways are not competing alternatives, but complementary approaches rooted in different mechanisms and tailored to various clinical constraints. However, it should be emphasized that these 2 pathways are currently at different stages of translation, with tethered interventions already established in clinical practice, whereas most untethered micro/nanorobotic systems remain at the preclinical or proof-of-concept stage. Together, they point toward a future in which multimodal synergy establishes a more efficient, precise, and safer paradigm for thrombus recanalization. In parallel, intelligent micro/nanomotors have increasingly been explored as active imaging agents capable of real-time tracking, signal amplification, and multimodal imaging integration in complex biological environments, further supporting the development of imaging-guided therapeutic systems [27]. Accordingly, this review summarizes the technological evolution, principal advantages, and inherent constraints of both tethered and untethered strategies, and further explores their potential integration under unified imaging guidance. We highlight how the convergence of these modalities may overcome longstanding clinical bottlenecks and accelerate the translation of next-generation thrombolytic platforms. This review is a narrative, application-oriented synthesis rather than a formal systematic review. The literature discussed here was selected from representative studies and recent reviews on thrombolysis, catheter-based thrombectomy, micro/nanorobotic systems, and imaging-guided intervention, with particular emphasis on works that clarify mechanistic principles, translational relevance, and the potential integration of tethered and untethered strategies. Compared with prior reviews that mainly focus on untethered micro/nanorobotic systems, carrier design, or individual imaging modalities, the present review emphasizes the comparative and complementary roles of tethered and untethered strategies within a unified thrombus-recanalization framework. Within this framework, acute ischemic stroke (AIS) is mainly discussed as a representative arterial large-vessel occlusion setting, whereas pulmonary embolism (PE) and deep venous thrombosis (DVT) are used as representative venous thrombotic settings; preclinical flow and animal models are included primarily to illustrate emerging untethered design concepts rather than equivalent clinical maturity across vascular beds.

## Tethered Therapeutic Strategies

**Fig. 2.**
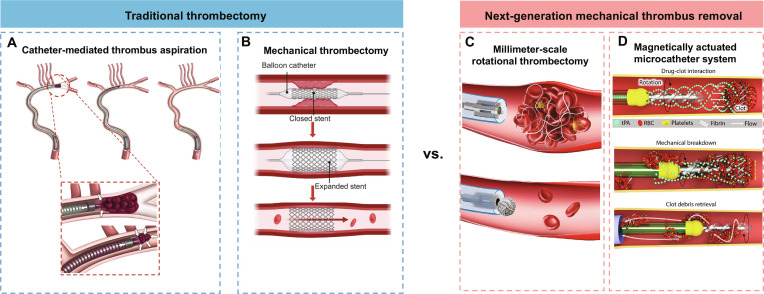
Comparison of conventional tethered thrombectomy modalities and representative next-generation tethered mechanical approaches. (A) The CANADAPT catheter removes thrombi via aspiration driven by externally applied negative pressure, typically relying on catheter lumen size and sustained suction to extract clot material [36]. (B) Schematic of stent retriever thrombectomy: the stent is deployed across the thrombus, embeds within the clot network, and is subsequently retrieved to remove the clot en bloc; performance can depend on clot composition/adhesion and may be associated with clot fragmentation. Figure created with BioRender.com. In contrast to these conventional mechanical strategies (A and B), which primarily rely on bulk extraction by suction and/or traction, (C and D) depict next-generation mechanical thrombectomy concepts that emphasize controlled local mechanical loading and actuation. (C) Millimeter-scale rotational thrombectomy, where coupled compressive and shear loading compacts the loose fibrin network, thereby reducing thrombus volume and facilitating removal [6]. (D) Operating principle of a magnetically actuated microcatheter system, enabling controlled distal motion and localized mechanical interaction with thrombi to improve maneuverability and targeted action in tortuous vasculature [49].

The principal advantage of tethered endovascular interventions lies in their ability to provide stable positioning and reliable force transmission, thereby enabling precisely localized recanalization procedures. These features make tethered approaches particularly suitable for proximal large-vessel occlusions or lesions with a high thrombus burden that require staged debulking strategies [28–30]. Over the past decades, the technological landscape of tethered interventions has evolved from early single-modality techniques, such as stent retriever thrombectomy (Fig. 2B) or direct aspiration (Fig. 2A), to a modular and combination-oriented pattern. In this framework, mechanical thrombectomy serves as the core component, while adjunctive local thrombolysis and energy-assisted modalities are selectively incorporated according to lesion characteristics and patient-specific risk profiles. For occlusions in large arterial vessels (e.g., acute ischemic stroke), aspiration and stent-based methods, either alone or in combination, remain first-line strategies, while adjunctive intra-arterial thrombolysis is still under active clinical evaluation. In venous thrombosis and pulmonary embolism, combined pharmacological thrombolysis and mechanical debulking, often aided by energy-enhanced CDT such as ultrasound-assisted systems, are commonly used to improve drug penetration and clot clearance. Throughout these procedures, multimodal imaging, including digital subtraction angiography (DSA), ultrasound, computed tomography (CT), and magnetic resonance imaging (MRI), play an essential role in lesion localization, procedural calibration, and complication monitoring. As a result, a modular therapeutic toolbox with imaging guidance has become a defining direction for the future development of tethered endovascular interventions [31–35].

### Mechanical thrombectomy

Mechanical thrombectomy achieves rapid thrombus debulking primarily through 2 technical routes. The first is stent retriever-based thrombectomy, in which an expandable mesh device mechanically engages the thrombus and is subsequently withdrawn. The second relies on negative-pressure aspiration, such as a direct aspiration first-pass technique (ADAPT), in which a large-bore aspiration catheter is advanced to the proximal face of the thrombus and continuous suction is applied for clot removal [36–38]. Taking aspiration-based strategies as an example, Lui and colleagues proposed the CANADAPT protocol, which refined ADAPT from an experience-driven maneuver into a reproducible 3-step workflow. Step 1 involves face-on aspiration at the thrombus surface. Step 2 maintains catheter engagement while withdrawing to a patent vessel segment with continued aspiration. Step 3 retracts the system into the guide catheter and completes clot removal with manual aspiration. The core principle of this approach lies in the synergy between negative suction force and arterial systolic pressure. Controlled catheter withdrawal under sustained aspiration enhances circumferential pressure acting on the thrombus, thereby promoting clot entry into the catheter lumen. Such a procedure can not only improve recanalization efficiency but also facilitate rapid angiographic reassessment and procedural rescue when needed [39] (Fig. 2A). Building on these advances, aspiration thrombectomy has recently been expanded to treat dense, treatment-resistant thrombi. Zhao and coworkers [6] introduced milli-spinner thrombectomy coupling a millimeter-scale hollow rotating head, which can be delivered through standard catheters. When rotated at high speed near the proximal surface of the thrombus, the device induces localized fluid recirculation, generating negative pressure that draws the thrombus tightly against the device tip. Simultaneously, combined compressive and shear loading compacts the fibrin network and expels red blood cells, reducing thrombus volume via microstructural remodeling rather than conventional tearing or cutting (Fig. 2C). This mechanism can reduce thrombus volume to approximately 10% of its original size; consequently, the remaining dense fibrin core becomes easier to capture and remove. Validation in vitro pulmonary and cerebral artery models, as well as in vivo porcine studies, demonstrated ultrafast debulking and high-fidelity recanalization while potentially lowering the risk of distal embolization associated with thrombus fragmentation.

Overall, stent retriever thrombectomy and direct aspiration demonstrate comparable efficacy at the population level. In clinical practice, selection between these approaches is driven more by vascular anatomy (e.g., vessel tortuosity, occlusion length, and proximal support) and by thrombus characteristics, including viscoelasticity and composition. Softer, red blood cell-rich clots are generally more amenable to aspiration thrombectomy, whereas denser, fibrin-rich thrombi often require greater radial support and mesh-based engagement to achieve effective capture. To improve first-pass success rates and reduce the incidence of distal embolization, combined strategies have been widely adopted. These strategies include synchronized proximal aspiration during stent retriever withdrawal and proximal flow arrest, both intended to minimize thrombus detachment and fragmentation during device retrieval. At the same time, a growing class of rotational and shear-based distal tools has emerged to address dense and treatment-resistant thrombi. By applying localized shear forces and compressive rotation action, these devices enhance clot removal efficiency while limiting frictional injury to the vessel wall. For example, Zhang et al. [40] introduced a vortex ultrasound-assisted endovascular sonothrombolysis system for cerebral venous sinus thrombosis. This approach integrates a forward-viewing transducer array at the catheter tip to generate vortex ultrasound, thereby inducing rotational flow, localized shear stress, and recirculation around the thrombus. In the reported in vitro 3-dimensional (3D) cerebral venous sinus thrombosis (CVST) model, these effects enabled rapid clot disruption and recanalization, while safety evaluation showed no evident vessel wall damage in ex vivo canine veins and a predominantly sub-100-μm debris profile under the tested conditions.

### Catheter-directed thrombolysis

CDT delivers fibrinolytic agents into or adjacent to the thrombus through multi-side-hole infusion catheters, creating a high local concentration gradient that increases intrathrombus drug exposure and improves therapeutic efficacy while allowing substantially lower doses than systemic thrombolysis [41–43]. CDT is therefore widely applied in iliac–femoral venous thrombosis, selected deep vein thrombosis, and intermediate-to-high-risk pulmonary embolism [2,9–11]. Its clinical value lies in the ability to raise the effective drug concentration and contact time within the thrombus without markedly increasing the risk of systemic bleeding. However, simple continuous infusion remains constrained by the solidified structure of the thrombus and by limited convective transport, which often leads to poor drug penetration, particularly in chronic, dense, or fibrotic thrombi. Consequently, recent advances in CDT have emphasized device-assisted thrombus modification to enhance local drug transport, forming a composite workflow of “restructure or loosen first, then dissolve”. Approaches include gentle mechanical agitation, localized separation of the thrombus, or reconstruction of microchannels to improve intrathrombus convection and drug penetration. In complex cases, sequential strategies combining mechanical debulking followed by short-term local thrombolysis are frequently adopted. The rationale is to rapidly reduce the proximal thrombus burden and restore perfusion, followed by a brief pharmacological phase to address residual thrombus and distal micro-emboli, thereby balancing efficacy and safety. A representative example is the Bashir multi-side-hole catheter system. Its distally expandable mesh structure can locally separate dense thrombus and create new infusion pathways. When combined with local thrombolysis in acute pulmonary embolism, this approach has been shown to reduce the right-to-left ventricular diameter ratio and improve pulmonary circulatory obstruction indices [18].

### Energy- and field-assisted interventions

The key concept is to overcome the limitation of poor drug penetration with controllable enhancement. Tethered platforms possess the unique advantage of delivering external energy through the same catheter pathway, with real-time imaging guidance to define safe boundaries for power, dosage, and duration [44–49]. Among these approaches, ultrasound-assisted catheter-directed thrombolysis (USAT) is one of the most clinically established techniques [49,50]. It promotes intrathrombus dispersion and penetration of fibrinolytic agents through acoustic vibration and microstreaming effects. The EkoSonic (EKOS) platform is a representative system for USAT. In this design, a coaxial ultrasound core is integrated within a 5.4-Fr infusion catheter to deliver low-energy, noncavitational ultrasound. The acoustic field loosens and unravels fibrin bundles, thereby improving the transport of recombinant tissue plasminogen activator (rt-PA) into the thrombus. The control unit continuously monitors acoustic power and catheter temperature, while the standardized clinical protocol delivers local rt-PA infusion via femoral venous access, often using bilateral catheter placement. A commonly used regimen is approximately 10 mg of rt-PA per catheter over about 15 h, with dose reduction in patients at higher bleeding risk. In the Bern Acute Pulmonary Embolism Registry (ERASE PE) registry cohort reported by Draxler et al. [51], the mean cumulative rt-PA dose per patient was 19.8 mg ± 6.6 mg over 14.4 h ± 2.2 h. Overall, 88% of patients experienced a reduction in mean pulmonary arterial pressure of 8.5 mmHg ± 7.7 mmHg, and the right-to-left ventricular diameter ratio decreased from 1.41 to 1.03. The in-hospital mortality rate was 3.2%, and the overall bleeding rate was 7.9%, including an intracranial hemorrhage rate of 1.0%. These findings indicate that combining ultrasound with low-dose local thrombolysis can improve right ventricular load while maintaining a relatively acceptable safety profile (clinical).

Building on these approaches, more intensive acoustic modalities, such as therapeutic cavitation ultrasound, are being explored to enable controlled mechanical disruption of thrombi and further shorten treatment duration. In parallel, hybrid strategies that combine magnetically responsive and acoustically responsive carriers are being developed to target micro-embolic fragments that remain after thrombectomy or thrombolysis. For example, Li et al. [52] employed targeted magnetic microbubbles (TMMBs) together with an interventionally and magnetically steerable catheter to actively capture and remove freely circulating microthrombus fragments. Their study demonstrated that magnetic microbubbles can be rapidly enriched by the magnetic field and adhere to thrombus debris within flowing blood, thereby markedly reducing the burden of circulating microthrombi and improving downstream microvascular perfusion. By concentrating, capturing, and retrieving these fragments, this work provides a novel interventional strategy for addressing the “no-reflow” phenomenon caused by microembolization following thrombectomy or thrombolysis. Overall, the goal of energy enhancement is not to maximize power output but to define a reproducible and imaging-guided parameter window that specifies safe and effective combinations of power, dosage, and duration. This framework ensures that therapeutic amplification and tissue safety remain simultaneously controllable. For illustration, representative operating conditions and associated safety readouts reported for selected enhancement modalities are summarized in Table 1. In addition, Zhang et al. [53] has advanced this field by focusing on distal device-end structural design. They developed the magnetically actuated soft rotating microcatheter (MSRM), which incorporates a magnetic-responsive soft rotary element at the microcatheter tip (Fig. 2D). With magnetic actuation, the system enables remote directional control and rotation-assisted steering to markedly improve access and navigation efficiency within narrow and tortuous distal vessels. After reaching the target site, the device can perform localized drug delivery, gentle mechanical interaction, and retrieval of thrombus fragments without the need for device exchanges, finally improving overall procedural efficiency. Its performance has been validated in in vitro vascular models and in in vivo rabbit studies. In addition, tests in human placental vessels indicate a low risk of vessel wall injury, reflecting that the combination of soft material with conservative actuation can well balance efficacy and safety (in vitro and in vivo preclinical). Although most of these studies remain at the preliminary validation stage, they collectively outline a clear translational trajectory. By using the catheter as a delivery conduit and integrating external energy fields with intelligent carriers or distal functional structures, hybrid CDT platforms have the potential to serve as a critical bridge between conventional tethered interventions and the emerging era of micro- and nanorobotic therapies.

**Table 1.** Representative operating conditions and major safety readouts of selected enhancement modalities for thrombus intervention

Modality	Representative operating conditions	Main safety readout(s)/limiting factors
Catheter-based USAT/EKOS	Reduced-dose, shortened-infusion regimens have been clinically explored, e.g., 10–20 mg over ~15 h or 4–12 mg/lung over 2–6 h [96,97]	Major/minor bleeding Intracranial hemorrhage Dose–duration balance under clinical risk constraints
Ultrasound-assisted/cavitation-enhanced systems	Representative reported parameters include kHz-range low-frequency ultrasound, defined acoustic intensity, MI, and exposure duration (e.g., continuous treatment for tens of minutes) [48]	Excessive cavitation Thermal rise Endothelial/vessel wall injury Hemorrhagic risk
Magnetic actuation/microswarms	Representative actuation conditions include mT-range magnetic field strength and Hz-range rotation frequency [98]	Residual retention Retrieval/termination efficiency Mechanical loading on vessel wall Biodistribution and organ accumulation
Optical/photothermal systems	Representative NIR-triggered systems commonly use 808-nm irradiation with controlled power density and exposure time [84]	Thermal injury Hemolysis/hemocompatibility concerns Off-target heating Organ distribution/long-term accumulation
Self-propelled/enzyme-driven micromotors	Propulsion is typically supported by endogenous or local biochemical fuels; quantitative operating windows are often less clearly standardized than in field-driven systems [99]	Fuel variability Byproduct-related biosafety concerns Limited control over termination Incomplete safety quantification

### Tethered interventional strategies: Advantages, limitations, and integration with micro- and nanotechnologies

Tethered endovascular interventions offer obvious advantages in terms of procedural maturity, device controllability, and regulatory compatibility. However, their therapeutic boundaries are constrained by anatomical accessibility and limited microscale maneuverability. Small-caliber distal vessels and microcirculatory lesions remain difficult to reach directly, and residual micro-emboli as well as dense or chronic thrombi that are insensitive to fibrinolysis often cannot be fully resolved within the available therapeutic window. These challenges define an inevitable pathway for technological integration. In this approach, the catheter provides proximal localization, delivery of energy and/or drugs, and procedural redundancy while releasing micro- or nanoscale agents to reach distal thrombi beyond the catheter’s range, enabling targeted clot removal and microenvironment modulation. For example, Wang et al. [54] demonstrated a programmable colloidal micro-swarm system that can be remotely actuated by an external magnetic field. This system is capable of rapidly approaching and detaching adherent thrombi within blood vessels and achieves directed transport and catheter-based retrieval of the debris via controllable magnetization by remotely stratifying and magnetizing thrombus fragments. The system facilitates rapid clearance under magnetic field actuation. This approach enables rapid vessel recanalization while minimizing distal embolization-induced microcirculatory obstruction, and is therefore promising for fast, safe, and efficient interventional thrombectomy. Zhang et al. demonstrated this concept using approximately 300 nm Fe_3_O_4_@mSiO_2_ nanorobots functionalized with tissue plasminogen activator (tPA) and preloaded within the catheter lumen. After the catheter reaches a bifurcation region, temporary flow arrest is realized with a balloon catheter, and the nanorobots are released under real-time fluoroscopic guidance. An externally applied rotating magnetic field assembles them into a navigable colloidal microswarm capable of reaching thrombi in submillimeter distal branches such as M3 or M4 vessels. The system achieves efficient recanalization through the combined effect of magnetically induced rolling and stirring that provides mechanical disruption, together with localized tPA-driven biochemical thrombolysis. After treatment, the microswarm is guided back to the catheter tip and aspirated for removal, forming an integrated workflow of precise delivery, localized amplification, and retrievable termination. At the same time, this workflow depends on temporary flow arrest, precise field-guided assembly/navigation, and reliable post-treatment aspiration, which may increase procedural complexity and still require further validation with respect to clinical feasibility in tortuous or time-sensitive settings. Results from animal studies indicate that this strategy shows the potential to improve distal recanalization efficiency while reducing systemic tPA exposure and shortening the overall thrombolysis duration (in vivo preclinical) [55].

## Untethered Therapeutic Strategies

In this review, “untethered systems” broadly refer to therapeutic micro/nanoscale agents delivered without continuous catheter or wire connection, including both injectable carrier-based platforms and actively actuated micro/nanorobotic systems. Introduced into the bloodstream as functional micro/nanoscale units, these untethered approaches may enable in vivo accumulation, deep penetration, and on-demand treatment through molecular targeting, microenvironment responsiveness, or activation by external fields such as magnetic, acoustic, or optical stimuli. Carrier-based systems mainly emphasize drug loading, targeting, and controlled release, whereas micro/nanorobotic systems further introduce active motion, external actuation, or programmable collective behavior. Together, they offer more precise intervention options for distal small vessels and microcirculatory lesions that are difficult to access using catheter-based techniques [56–59].

By contrast, tethered interventions derive operational certainty from catheter access, enabling precise localization, force application, and device retrieval. They are therefore more suitable for rapid recanalization of proximal large vessels or lesions with a high thrombus burden. Untethered systems, on the other hand, are intended to complement these limitations by offering microscale accessibility, active transport, and localized therapeutic amplification. Collectively, these 2 paradigms are not competing alternatives but complementary strategies: Tethered approaches primarily enable pathway-based macroscopic recanalization, whereas untethered systems offer microscopic precision and distal reach.

**Fig. 3.**
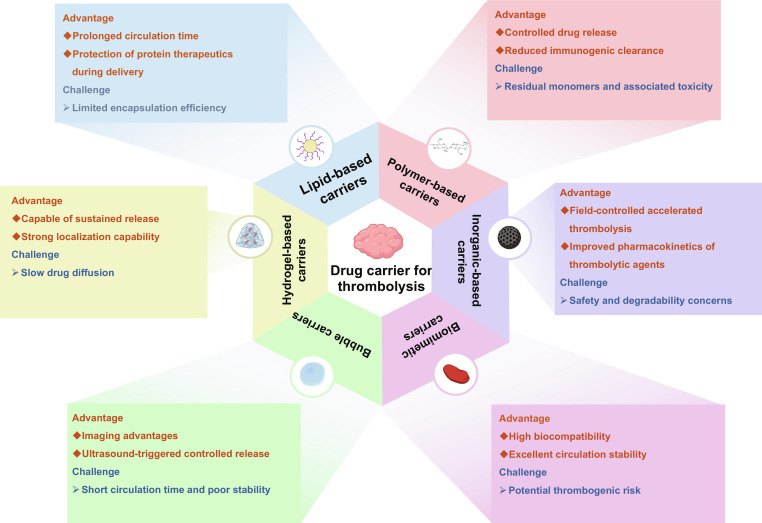
Representative drug-carrier platforms for thrombolysis. Schematic overview of major carrier classes (e.g., lipid-, polymer-, hydrogel-, inorganic-, biomimetic-, and bubble-based systems) developed to improve thrombolytic drug delivery and clot dissolution. In general, these platforms aim to enhance local accumulation at thrombi, prolong circulation, and enable controlled or stimuli-responsive release to reduce systemic exposure and bleeding risk. Each carrier class offers distinct strengths while also presenting trade-offs (e.g., loading/protection, release controllability, targeting/field responsiveness, biocompatibility and safety, and in vivo stability); therefore, only a high-level summary is provided here without detailed discussion. Figure created with BioRender.com.

Within this untethered framework, carrier-based thrombolytic delivery platforms can be broadly classified into 6 major categories: lipid-based carriers, polymer-based carriers, inorganic and nanomaterial-based carriers, biologically derived or biomimetic carriers, hydrogel-based carriers, and bubble-based or acoustic carriers (Fig. 3). The following sections adopt this classification to explain how each carrier type achieves targeted accumulation and intrathrombus retention through structural and interfacial design. In addition, their coupling with external energy-based triggering or amplification—such as magnetic navigation, ultrasound-induced cavitation and microstreaming, and photothermal or photodynamic effects—is discussed to illustrate the development of synergistic thrombolysis strategies.

For rapid cross-comparison, the key features of the 6 major thrombolytic carrier classes are summarized in Table 2. These carrier classes differ in delivery format, targeting or actuation strategy, imaging compatibility, post-therapy fate, and translational limitations.

**Table 2.** Comparative overview of 6 major thrombolytic carrier classes for untethered therapy.

Carrier class	Format	Targeting/actuation	Imaging	Post-therapy fate	Safety/limitations
Lipid-based carriers [62]	Liposomes, lipid vesicles	Ligand targeting; adhesion; stimulus-responsive release	Usually needs added labels	Biodegradation; metabolic clearance	Limited shear stability; shallow penetration
Polymer-based carriers [63]	Nanogels, porous particles, polymer networks	ROS/thrombin/pH-responsive release; programmable delivery	Can load imaging agents	Composition-dependent degradation and clearance	Limited fibrin penetration; off-target release
Inorganic/nanomaterial-based carriers [64]	Magnetic, optical, acoustic, catalytic nanomaterials	Magnetic, acoustic, or optical activation	Often suitable for multimodal imaging	Composition-dependent clearance; possible retention	Accumulation risk; material-related toxicity
Biologically derived/biomimetic carriers [65–68]	Membrane-coated particles, cell-inspired vesicles	Bioadhesion; immune evasion; thrombus affinity	Usually needs external labeling	Biodegradation; reticuloendothelial clearance	Off-target retention; uncertain long-term fate
Hydrogel-based carriers [69]	Injectable gels, local depots	Local retention; sustained release	Usually limited unless labeled	Controlled degradation or local persistence	Dislodgement risk; difficult retrieval
Bubble-based/acoustic carriers [70]	Microbubbles, nanobubbles	Ultrasound-triggered release; cavitation; microstreaming	Intrinsically ultrasound-visible	Bubble collapse; gas dissipation; carrier clearance	Acoustic sensitivity; cavitation safety window

### Categories and selection of drug carriers

The ability of a drug carrier to circulate stably under high-shear blood flow, adhere effectively to the thrombus surface, penetrate the dense fibrin network, and achieve on-demand drug release with enhanced therapeutic activity is determined by material properties and structural design [60,61]. This section analyzes 6 major classes of drug carriers and summarizes their advances and prospects.

Lipid-based carriers leveraging membrane-like interfacial properties offer excellent biocompatibility and can be readily engineered through surface modification, which facilitates the incorporation of targeting ligands or stealth layers. These features allow lipid carriers to maintain stability in the bloodstream while simultaneously promoting adhesion to thrombotic lesions. For example, Jin et al. [62] developed PEGylated liposomes to prolong systemic exposure of streptokinase (SK) and mitigate its rapid clearance. Compared with free SK or non-PEGylated liposomes, the PEGylated liposomes extended the circulation time of SK and increased the overall drug-contact duration in vivo. Nevertheless, the structural stability and release consistency of lipid-based systems under high shear stress and in protein-rich biological environments remain critical challenges. In addition, without proper morphological and transport design, conventional micro/nanoparticles are confined to the thrombus surface rather than penetrating into its interior. This limitation explains why current advances in lipid-based thrombolytic carriers increasingly rely on coupling with external field triggering or active motion strategies to overcome transport barriers and improve intrathrombus delivery.

By comparison, polymer-based carriers offer distinct advantages in structural design. Tubular, porous, layered, and degradable network architectures not only enhance drug-loading capacity but also provide pathways for microenvironment-responsive and programmed drug release. These systems can be engineered to sense pathological cues such as reactive oxygen species (ROS), thrombin, or pH, and trigger site-specific payload release (e.g., via disassembly or pore opening) while remaining relatively stable in normal regions. Furthermore, segmented or multilayer architectures allow sequential release patterns and coordinated multi-drug delivery. A representative example is the ROS-responsive sulfated polysaccharide nanogel developed by Cao et al. [63]. By incorporating ROS-sensitive crosslinking sites within a 3D polymer network, the nanogel gradually degrades and releases its cargo in regions of elevated ROS levels. At the same time, polymer acts as a sponge to scavenge excess ROS, thereby providing an integrated therapeutic effect by combining microenvironment modulation with localized drug release. However, relying solely on chemical responsiveness often makes it difficult to overcome the physical barrier posed by the dense fibrin network of thrombi. As a result, current research attempts to integrate external energy inputs or mechanical perturbation to enhance transport, addressing the limitation of poor penetration.

Inorganic and nanomaterial-based carriers offer a different design paradigm by directly converting intrinsic material properties into therapeutic advantages rather than functioning solely as drug vehicles. Ordered crystalline frameworks, porous architectures, and specific crystal phases can mediate energy conversion or catalytic reactions under magnetic, acoustic, or optical stimulation, thereby contributing to thrombus removal and modulation of the local pathological microenvironment. For example, Xu et al. [64] developed a magnetically responsive platform for the clearance of reactive oxygen and nitrogen species. By incorporating both metal ions and bioactive ions into a nanostructured framework, the system adjusts the local redox environment under an external magnetic field, achieving a stepwise therapeutic sequence in which microenvironment restoration precedes enhanced thrombolysis. Such inorganic and nanomaterial carriers can amplify therapeutic outcomes even with reduced reliance on conventional thrombolytic drug loading. However, they also present challenges related to metabolic clearance, long-term accumulation, and potential toxicity. Consequently, the intrinsic biocompatibility and degradability of these materials are now viewed as key prerequisites for future clinical translation.

Biologically derived and biomimetic carriers share a common design principle that relies on reprogramming interfacial behavior by borrowing the innate function of blood cells. Red blood cell membranes provide advantages in prolonged circulation and immune evasion, whereas platelet membranes offer strong adhesion to damaged endothelium and thrombus surfaces. As a result, core–shell architectures constructed by cell membranes can simultaneously confer stealth properties and site-specific adhesion. A typical strategy involves coating diverse core structures, such as hollow mesoporous Prussian blue or polymeric nanoparticles, with platelet membranes. This approach ensures effective deliverability while presenting platelet-like surface receptors that enhance affinity for fibrin-rich thrombotic regions [65,66]. Zhang and colleagues [67] further demonstrated that tuning the size, shape, and deformability of platelet-inspired particles enables simultaneous adhesion and mechanical adaptability within heterogeneous thrombus environments. This dual capability allows the carriers to navigate local constrictions and shear gradients while contributing to both thrombolysis and endothelial repair. Similarly, Quan et al. [68] engineered annexin V onto the surface of nanoscale systems to achieve a controllable adhesion switch within pathological microenvironments by employing its affinity for phosphatidylserine exposure. Despite these advantages, biologically derived and biomimetic carriers face an inherent trade-off: Enhanced adhesion promotes targeted accumulation but may also increase the risk of off-target retention. Therefore, current research directions emphasize more precisely engineered membrane interfaces and rigorous characterization of in vivo biodistribution, clearance, and long-term fate to ensure both efficacy and safety.

When the therapeutic goal extends from merely reaching the thrombus to sustaining activity, hydrogel-based systems function more as local drug reservoirs rather than transport vehicles. By maintaining a high local drug concentration and providing controlled release near the lesion, these systems can reduce systemic exposure and prolong the therapeutic window. They are particularly well suited in scenarios that require localized coverage or in patients for whom minimizing systemic bleeding risk is a key priority. In a representative study, Glazier et al. [69] employed urokinase-coated hydrogel balloons to treat patients with angiographically visible coronary thrombi. This approach enables contact-based, site-specific thrombolysis within the coronary artery and can be seamlessly integrated into interventional recanalization procedures. Conceptually, this approach transforms systemic drug administration into device-centered, localized delivery in which the instrument itself acts as a controlled drug depot. However, the applicability of hydrogel-based systems is highly dependent on the accessibility and stability of the delivery site. In the dynamic vascular environment, characterized by strong shear forces and spatial constraints, maintaining in situ stability, preventing displacement, and enabling safe retrieval remain critical engineering challenges.Addressing these constraints is essential for the translation of hydrogel-based local delivery platforms.

Bubble-based and acoustically responsive carriers are another representative delivery system. Under acoustic excitation, microbubbles and nanobubbles can generate oscillation, cavitation, or microjets that produce localized high shear forces and microstreaming. These effects physically loosen the fibrin network, promote drug release, and enhance convective transport and diffusion within the thrombus. Importantly, these carriers also provide an intrinsic imaging interface, enabling the feedback loop that integrates localization, activation, and real-time evaluation. A representative example is RGDS-targeted microbubbles loaded with urokinase employing diagnostic ultrasound to activate the system. This approach exemplifies the coordinated pathway of targeted accumulation, ultrasound triggering, and synchronized drug release with enhanced intrathrombus penetration [70]. It is important to note that the therapeutic performance of these systems is highly sensitive to acoustic parameters, tissue-specific attenuation, and interindividual variability. Moreover, cavitation must be strictly confined within a defined safety window in order to avoid off-target tissue injury.

Taken together, those drug carriers tackle different bottlenecks in thrombolytic delivery, such as circulation stability, thrombus targeting/adhesion, fibrin penetration, and spatiotemporally controlled drug release. Lipid-based systems offer advantages in biocompatibility and surface engineering. Polymer-based platforms offer greater flexibility in structural programmability and stimulus-responsive release. Inorganic and nanomaterial systems harness intrinsic physical properties to enhance the therapeutic effect. Biomimetic carriers leverage biological mechanisms to improve accumulation and retention. Hydrogel-based systems function as localized drug reservoirs that provide sustained release. Bubble-based systems are highly compatible with ultrasound triggering and closed-loop imaging feedback. Despite these distinctions, they share a common limitation: the penetration barrier posed by dense thrombi and the difficulty of achieving reliable, controllable behavior in vivo. Consequently, current carrier design strategies are shifting from simple drug transport toward integrated platforms that couple carriers with external energy inputs and imaging guidance, thereby overcoming the limitation of penetration and controllability.

### Synergistic strategies for effect amplification and drug delivery

Across the 6 carrier categories described above, the material platform determines biocompatibility, drug-loading capability, and stimulus responsiveness. However, the therapeutic performance in thrombus treatment is still restricted by the microstructural and hemodynamic barriers of the lesion. Dense fibrin networks possess small pore sizes and high resistance to penetration, which makes passively diffusing particles prone to remaining on the clot surface rather than achieving deep intrathrombus delivery. Blood flow shear and rethrombosis risk further limit delivery efficiency and long-term therapeutic outcomes. Consequently, the field has gradually shifted from focusing solely on the materials selection toward understanding how structural design and energy-assisted mechanisms can be combined to enhance drug delivery and amplify therapeutic effects.

**Fig. 4.**
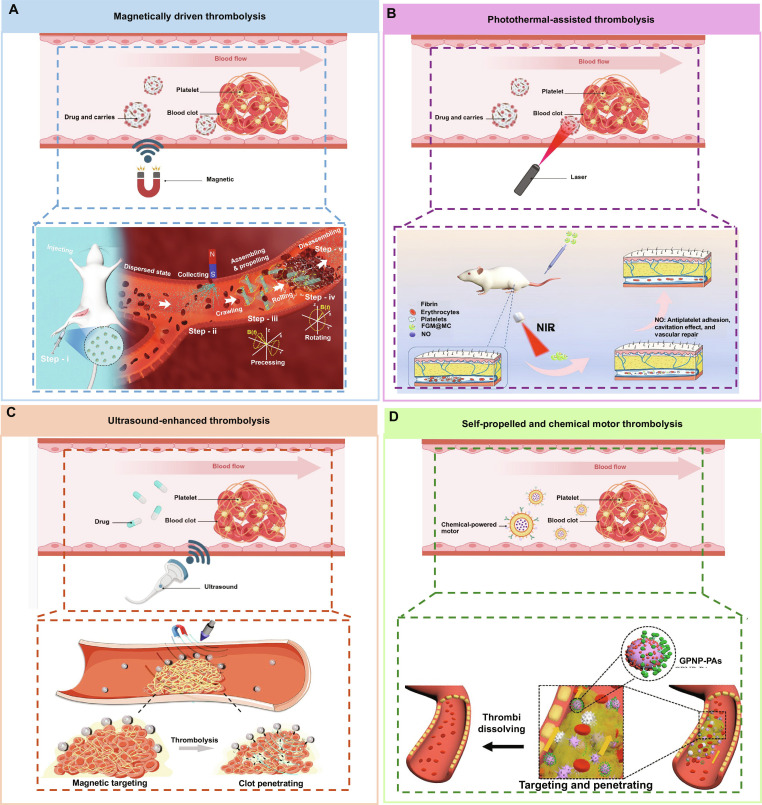
Untethered combinatorial strategies for thrombolytic therapy. Schematic summary of 4 representative mechanisms that enhance thrombolysis via external fields or autonomous motion: magnetic field-guided targeting and mechanical agitation (A), optical/near-infrared (NIR) photothermal heating to accelerate clot microstructural loosening and drug penetration (B), acoustic (ultrasound) enhancement for localization, triggered release, and improved intraclotal transport (C), and self-propelled chemical/micromotor action enabling active penetration and thrombus-directed accumulation (D). (A) Heparinoid polymer brush-grafted magnetic nanorobots (HPB-NRs) based on MB@PSS nanoparticles, highlighting biosafety, drug-loading capacity, and magnetically targeted thrombolysis [80]. (B) Photothermal-assisted thrombolysis enabled by fucoidan/S-nitrosoglutathione/melanin@microcapsules (FGM@MCs) under NIR irradiation [81]. (C) Precision delivery combining magnetic localization with ultrasound-triggered tPA release from magnetic microbubbles coated with SiO_2_ and loaded with tPA (MMB–SiO_2_–tPA) [71]. (D) Self-propelled targeted thrombolysis mediated by GOx-powered Janus platelet nanoparticles (GPNP–PAs) through autonomous locomotion and thrombus-directed accumulation [82]. Figure created with BioRender.com.

Regarding the structural design, anisotropic geometries such as disc-like, rod-like, helical, Janus, or bowl-shaped designs can reduce hydrodynamic resistance by wall-following motion, rolling, or peristaltic-like propulsion. These features improve transport efficiency in the endovascular milieu and provide a structural basis for deep penetration under external field activation. Beyond geometry, different external fields offer distinct physical mechanisms for penetration and therapeutic amplification. Magnetically actuated systems combine targeted accumulation with torque-driven motion, enabling repeated pulling and stirring within the thrombus. This process can remodel fibrin pore architecture and expand diffusion pathways for drug transport (Fig. 4A). Acoustically driven systems rely on cavitation and microjet formation to generate localized high shear forces, achieving coupled amplification through physical loosening of the clot together with synchronized drug release and enhanced convective transport (Fig. 4C). Light-driven systems induce localized heating that softens the thrombus network and promotes convective diffusion, making them suitable for superficial lesions or sites with accessible optical stimuli (Fig. 4B). In addition, self-propelled systems and chemical micromotors generate propulsion by exploiting substrate gradients, offering the potential to operate without excessive intervention. However, key translational challenges remain, including the safety of reaction byproducts such as peroxides, motion stability in vivo, and the controllable termination and clearance after treatment (Fig. 4D).

Notably, the evaluation of active motion should not rely solely on speed, as the therapeutic task-related performance is more important: the ability to reach and remain at the clot, penetrate the fibrin network, deliver a sufficient drug dose, and ultimately realize recanalization with acceptable bleeding risk. Under blood flow disturbances and within complex thrombus networks, the key questions are whether carriers can achieve controllable approach, retention, and withdrawal. Based on this perspective, the following section presents representative exemplars of untethered systems that implement these amplification mechanisms under magnetic, optical, acoustic, or self-propelled modes.

### Representative exemplars of untethered systems for enhanced thrombolysis

Representative studies further illustrate how the amplification principles outlined above are implemented in specific untethered systems. Magnetic field-based strategies mainly exploit field-guided assembly and collective agitation to enhance targeted accumulation and mechanical interaction with thrombi [71–74]. Optical strategies rely on spatially and temporally controllable energy deposition to induce local heating and improve drug penetration [75–79]. Acoustic strategies integrate activation with enhanced intrathrombus transport, often through oscillation, microstreaming, or related mechanical effects [60,80–83]. Self-propelled systems use chemical propulsion to overcome the limitations of passive diffusion in complex flow environments and to facilitate penetration into dense fibrin networks [84–88]. For magnetic field-based strategies, Yang et al. [89] developed heparinoid-like polymer brushes grafted onto magnetic nanorobots to enhance biocompatibility. Under an external magnetic field, the particles assemble into a navigable swarm whose collective motion produces mechanical perturbation and localized accumulation, thereby enabling synergistic thrombolysis. Importantly, once the therapeutic task is completed, the swarm can be disassembled, reducing the risk of undesired retention in vivo (Fig. 4A). In optical field-based strategies, the therapeutic effect is partially transferred to structurally programmable photothermal units. Yang et al. [90] demonstrated a drug-free photothermal approach in which near-infrared irradiation enables localized energy deposition. Through the coupling of structural design and photothermal behavior, this system enables effective penetration and therapeutic action within dense fibrin networks (Fig. 4B); however, the clinical translation of optical strategies remains constrained by the attenuation and limited penetration of light in deep tissues. In contrast, acoustic field-based approaches offer the distinct advantage of imaging, activation, and real-time assessment in deep soft tissues. In a representative study demonstrated by Wang et al. [60], a nanoparticle-shelled microbubble platform was developed to accelerate the thrombolytic process. In this system, ultrasound-induced microbubble oscillation enables thrombolytic agents to be delivered into the interior of the clot in a precise manner (Fig. 4C); in other words, acoustic fields function not only as external activators but also as dynamic tools that drive drug transport toward deeper intrathrombus penetration. The GOx-powered Janus platelet nanomotor is a representative example of a self-propelled system for targeted thrombolysis [91] (Fig. 4D). In this design, glucose oxidase converts the chemical energy of endogenous substrates into active propulsion and local stirring, and the Janus architecture further promotes thrombus adhesion, thereby enhancing in situ retention under disturbed blood flow.

These 4 categories of drug-delivery systems offer distinct advantages for thrombus removal. Magnetic fields allow controllable accumulation and reversible swarm behavior. Optical fields enable spatially and temporally programmable energy deposition. Acoustic fields achieve enhancement in convection and penetration together with an integrated imaging interface. Self-propelled systems combine active motion and targeting within endogenous biochemical reactions. Despite these differences, those systems are transitioning from passive arrival to active approaching and ultimately achieving the amplification for therapeutic outcomes in a repeatable, quantifiable, and safe manner. Therefore, achieving precisely controllable targeting, retention, and termination under realistic blood flow conditions should be the key performance metric for the development of novel untethered drug delivery systems in the future.

Under physiological hemodynamic conditions, different micro/nanorobotic platforms show distinct strengths and limitations. Magnetic systems generally provide the best controllability for directional accumulation and retention under flow, acoustic systems are advantageous for deep-tissue actuation but remain sensitive to cavitation conditions and vessel geometry, optical systems offer precise local activation but are limited by tissue penetration, and self-propelled systems may enhance active penetration but still face challenges in motion stability and controllable termination in vivo. Therefore, performance under flow should be assessed not only by propulsion but also by retention, penetration, recanalization efficiency, and safety.

Importantly, the post-treatment fate of micro/nanorobotic systems is a critical issue that directly affects both biosafety and translational feasibility. After therapy, retrieval or degradation generally follows 3 main pathways, depending on system design. First, catheter-assisted or magnetically actuated systems may be actively recollected or aspirated once thrombolysis is completed, particularly when external control and localization are maintained. Second, fully untethered platforms may be engineered to disassemble into smaller components or undergo biodegradation through hydrolytic, enzymatic, or other physiologically responsive processes. Third, nanoscale or degradable components may rely on systemic clearance pathways, such as reticuloendothelial uptake or renal/hepatobiliary elimination, depending on their size, composition, and surface properties. At present, however, the lack of clearly defined and clinically validated post-treatment retrieval or clearance routes remains a major translational challenge for micro/nanorobotic thrombolytic systems.

## Imaging and Tracking

**Fig. 5.**
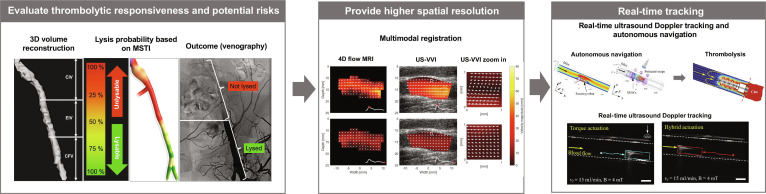
Imaging-driven closed-loop framework for thrombosis intervention. Imaging supports (i) thrombus localization and risk assessment [87], (ii) navigation of therapeutic devices or micro/nanoagents [88], and (iii) real-time therapy monitoring with feedback-regulated control [89].

Precise and safe thrombolysis requires a closed-loop framework in which imaging serves 3 core functions in both tethered and untethered systems: (a) localizing the thrombus; (b) guiding the navigation of therapeutic agents or devices; and (c) monitoring therapeutic response to enable feedback-based adjustment of therapy (Fig. 5). In this review, “closed-loop” refers to a system in which thrombus localization, device or agent guidance, and therapeutic monitoring are functionally linked to feedback-driven, real-time adjustment of treatment parameters on the basis of imaging-derived feedback. Clinically, ultrasound, DSA, and CT or MR angiography (MRA) are routinely used to identify the occlusion site, evaluate vascular access routes, and assess thrombus burden. In intraluminal settings such as coronary arteries, high-resolution modalities—particularly optical coherence tomography—can further resolve residual thrombus, endothelial injury, and stenotic morphology, thereby informing interventional decision-making [83,85,86]. Beyond localization, imaging also supports (a) navigation by integrating structural and hemodynamic information to improve spatial targeting accuracy in complex vascular geometries. Finally, (b) real-time tracking of thrombus reduction and device behavior enables adaptive adjustment of drug delivery or actuation parameters within safe operating limits, forming an imaging-driven closed-loop paradigm for thrombosis intervention.

**Fig. 6.**
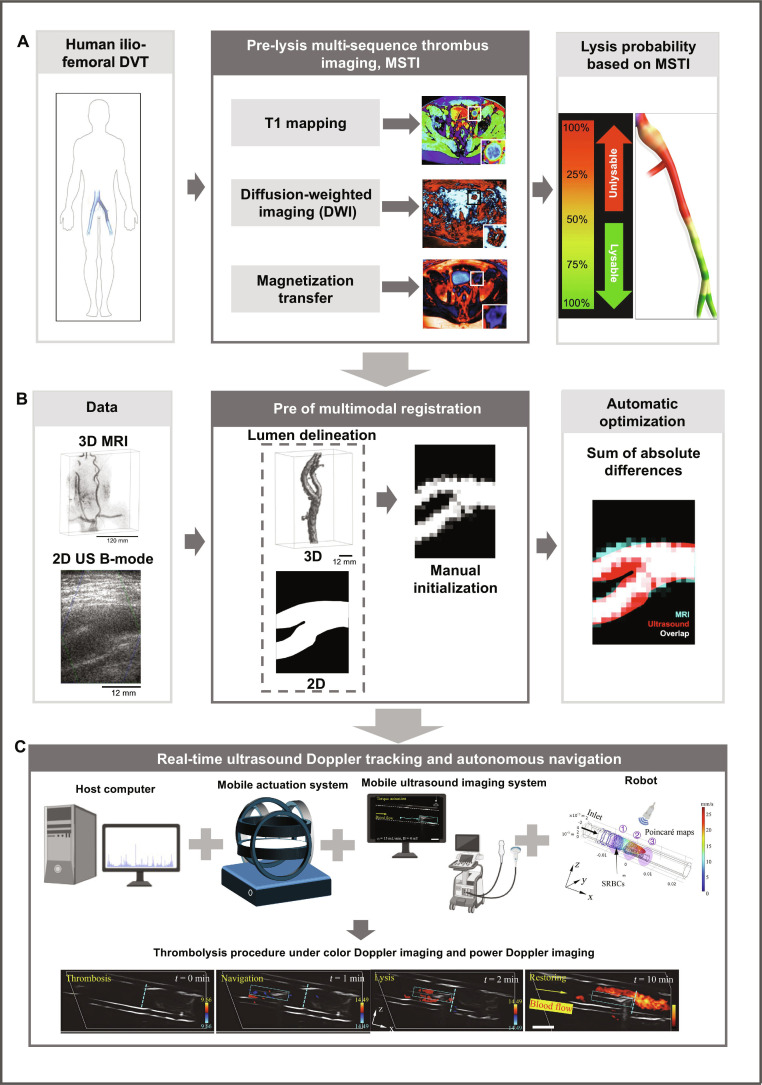
Closed-loop thrombus characterization, registration, and autonomous navigation framework. (A) Pre-lysis multi-sequence thrombus imaging (MSTI) combining T1 mapping, diffusion-weighted imaging/apparent diffusion coefficient (DWI/ADC), and magnetization transfer imaging to quantify thrombus composition and estimate lysis probability [87]. (B) Multimodal MRI–ultrasound registration aligns preoperative 3D vessel geometry with intraoperative 2D ultrasound through iterative spatial optimization, enabling accurate localization [88]. (C) A mobile actuation platform (hybrid torque–force control to counter blood flow) co-moves with a mobile ultrasound probe for real-time Doppler tracking, supporting closed-loop localization and autonomous navigation during thrombus intervention [89].

As imaging evolves from purely anatomical visualization to quantitative characterization, multiparametric MRI and vector-flow ultrasound provide insights into thrombus composition and local hemodynamics, enabling assessment of thrombolytic responsiveness and potential risk. These approaches offer objective metrics for tailoring treatment windows, dosing, and safety thresholds on a patient-specific basis. For example, Silickas et al. [92] reported a contrast-free multisequence thrombus imaging method (MSTI) that integrates 3D T1 mapping, 3D magnetization transfer imaging, and 2D diffusion-weighted imaging with apparent diffusion coefficient mapping. This combination allows detailed characterization of thrombus composition and stratification of its susceptibility to thrombolysis, thereby providing an imaging-based feedback for subsequent therapeutic strategy (clinical imaging study) (Fig. 6A).

In terms of tracking, tethered interventions emphasize visualized navigation and real-time monitoring of guidewires and catheters in order to reduce operator burden within complex vascular trees and improve procedural controllability. By contrast, untethered approaches prioritize quantitative assessment of the in vivo distribution, retention, penetration, and clearance of carriers or micro/nanorobots, as vascular complexity and flow dynamics strongly influence delivery efficiency and therapeutic performance. To address these needs, many studies achieved combination of localization, accumulation, activation, and evaluation by integrating photoacoustic imaging units, magnetic resonance, or CT contrast elements. Cross-modal image registration is also being explored to enable coordinated control of external field parameters and carrier trajectories. In addition, the high-frame-rate ultrasound velocity vector imaging technique proposed by Ruisch et al. [93] demonstrates the ability for capturing transient and small-scale recirculation zones. This capability provides a higher spatiotemporal resolution tool for understanding local risk mechanisms and delivery behaviors through detailed characterization of blood flow patterns (Fig. 6B).

The ultimate objective of imaging systems is to establish a closed-loop framework in which thrombus burden, blood flow restoration, and the spatial distribution of therapeutic units are converted into actionable feedback variables. These variables enable real-time adjustment of driving fields, energy dose, and drug delivery timing, ensuring that therapeutic amplification and safety remain simultaneously controllable. For example, Wang et al. [94] used Doppler ultrasound imaging to detect real-time imaging signals from robot rotation and provide positional feedback to the magnetic field control unit, enabling automated path planning and upstream navigation in branched vascular models. Furthermore, Doppler and B-mode imaging are simultaneously used to monitor thrombolysis progress (Fig. 6C). Overall, effective operation depends on real-time imaging with high signal-to-noise ratio and minimal tissue perturbation. Achieving this goal requires coordinated and integrated design ensuring imaging accessibility, robust contrast enhancement, and feedback-driven controllability in vivo.

## Summary and Outlook

Tethered endovascular interventions rely on established vascular access to achieve rapid vessel reopening and provide reliable device retrievability, making them the cornerstone for treating large-vessel occlusions. In contrast, untethered micro- and nanoscale systems enable distal accessibility, deep penetration, and therapeutic amplification, offering solutions for residual microemboli, microcirculatory obstruction, and dense or drug-resistant thrombi. Rather than serving as competing alternatives, synergistic integration is the optimal strategy. In this framework, tethered catheters assume responsibilities for localization, energy transmission, imaging surveillance, and procedural safety redundancy, while releasable micro- or nanoscale agents perform distal intervention and microenvironment modulation. This coordinated approach enables the combination of rapid proximal recanalization and distal microcirculatory restoration within a single therapeutic workflow, while drug dosage and energy parameters can be dynamically optimized according to real-time imaging feedback. However, several key barriers still limit clinical translation. Beyond thrombolytic efficacy itself, these include reliable navigation, retention, and controllable actuation under physiological hemodynamic conditions, as well as clearly defined post-therapy termination routes, ranging from active recollection and aspiration in catheter-assisted or magnetically actuated systems to disassembly, biodegradation, or systemic clearance in fully untethered platforms. Additional barriers include standardized safety windows for field-assisted intervention and practical integration with established imaging-guided interventional workflows. Together, these issues remain central obstacles to moving micro/nanorobotic thrombolytic systems from proof-of-concept studies toward clinically applicable platforms. Looking forward, thrombus-targeted recanalization technologies are likely to advance along 4 tightly coupled directions spanning tethered and untethered modalities. First, for tethered interventional systems (e.g., catheter-based or wire-guided platforms), priority should be given to enhancing intravascular maneuverability and procedural robustness, including atraumatic navigation in tortuous vessels, stable device–thrombus engagement under pulsatile flow, and reliable termination mechanisms—such as guaranteed retrieval, controlled deactivation, or bioresorbable designs—to minimize risks associated with unintended in vivo retention. Second, for untethered micro/nanoagents, the key need is programmable micro/nanomaterials capable of executing multi-stage functions in a prescribed sequence (adhesion, penetration, on-demand drug release, and local tissue protection) while reducing off-target exposure and improving spatiotemporal specificity in complex vascular microenvironments. Third, the field should move toward practical tethered–untethered cooperative systems, where tethered devices provide long-range access, accurate positioning, and fail-safe rescue control, while untethered agents enable distributed sensing and therapy in distal or hard-to-reach regions, thereby combining precision with coverage. Fourth, artificial intelligence (AI)-assisted control algorithms and imaging-driven closed-loop regulation should be further established by converting thrombus burden, agent distribution, and hemodynamic recovery into real-time quantitative metrics that support intraoperative assessment, adaptive actuation, and informed clinical decision-making. In parallel, recent perspectives on swarm autonomy have emphasized that the transition from simple collective actuation to machine-intelligent microswarm systems will be essential for future adaptive biomedical deployment [95]. As these advances converge, a unified software–hardware ecosystem is expected to emerge, integrating interoperable devices, real-time feedback, and intelligent control to enable recanalization strategies that are broader in reach, faster in response, more complete in efficacy, and safer for clinical translation.

## Data Availability

No new data were created or analyzed during this study. Data sharing is not applicable to this article.
